# Infectious titer determination of lentiviral vectors using a temporal immunological real-time imaging approach

**DOI:** 10.1371/journal.pone.0254739

**Published:** 2021-07-15

**Authors:** Jennifer J. Labisch, G. Philip Wiese, Kalpana Barnes, Franziska Bollmann, Karl Pflanz

**Affiliations:** 1 Lab Essentials Applications Development, Sartorius Stedim Biotech GmbH, Göttingen, Lower Saxony, Germany; 2 Institute of Technical Chemistry, Leibniz University Hannover, Hannover, Lower Saxony, Germany; 3 Faculty of Mathematics, Computer Science and Natural Sciences, RWTH Aachen University, Aachen, North Rhine-Westphalia, Germany; 4 BioAnalytics Applications, Essen BioScience, Royston, Hertfordshire, United Kingdom; 5 Segment Marketing Viral-based Therapeutics, Sartorius Stedim Biotech GmbH, Göttingen, Lower Saxony, Germany; Wake Forest School of Medicine: Wake Forest University School of Medicine, UNITED STATES

## Abstract

The analysis of the infectious titer of the lentiviral vector samples obtained during upstream and downstream processing is of major importance, however, also the most challenging method to be performed. Currently established methods like flow cytometry or qPCR lack the capability of enabling high throughput sample processing while they require a lot of manual handling. To address this limitation, we developed an immunological real-time imaging method to quantify the infectious titer of anti-CD19 CAR lentiviral vectors with a temporal readout using the Incucyte^®^ S3 live-cell analysis system. The infective titers determined with the Incucyte^®^ approach when compared with the flow cytometry-based assay had a lower standard deviation between replicates and a broader linear range. A major advantage of the method is the ability to obtain titer results in real-time, enabling an optimal readout time. The presented protocol significantly decreased labor and increased throughput. The ability of the assay to process high numbers of lentiviral samples in a high throughput manner was proven by performing a virus stability study, demonstrating the effects of temperature, salt, and shear stress on LV infectivity.

## Introduction

Most lentiviral vectors used for therapeutic applications are based on the human immunodeficiency virus (HIV) type 1 which belongs to the *Retroviridae* family and the genus *Lentivirus* [1]. Lentiviral vectors (LV) are efficient gene delivery vehicles playing an important role for advanced therapy medicinal products (ATMPs), that include gene therapy and gene-modified somatic cell therapy products [2]. The aim of ATMPs is to replace disease-causing mutated genes or to deliver a gene for the expression of therapeutic proteins. Lentiviral vectors represent the most frequently used viral gene delivery platform for the *ex vivo* generation of chimeric antigen receptor (CAR)-T cells for cancer immunotherapies [3]. Antigens with a high coverage on tumor cells are selected as targets for the CAR constructs to enhance T cell specificity [4]. CD19 is the most widely used target in CAR-T cell therapy to treat B cell lymphomas [5,6]. Five CAR-T cell therapy products are currently approved by the Food and Drug Administration, with Kymriah^™^, Breyanzi^®^, and Abecma^®^ relying on lentiviral vector-mediated gene transfer [7–9].

The increasing demand of lentiviral vectors due to the high gene-modified cell therapy and gene therapy market growth leads to supply shortfalls [10,11]. A significant bottleneck for viral vector process development and production is the vector quality control. To speed up the upstream and downstream development of lentiviral vector production process, reliable and efficient assays for their quantification are required. A method for fast and precise determination of lentiviral vector infectious titers is desperately needed for process development and process optimization, where typically a high number of samples are generated. Process development is decelerated by labor-intensive and time-consuming virus titer assays. Typically, virus quantification methods aim to determine either the total viral particle (VP) titer or the infectious virus particle titer given in transducing units (TU) per mL [11]. Infectious titer is more meaningful as it measures the number of virus particles that can infect target cells [12]. HEK293T cells are typically used as target cells for LV infectious titer determination [13–18]. The infectious titer of lentiviral vectors can be determined by transduction of cells followed by quantification of the proviral DNA copy number by quantitative polymerase chain reaction (qPCR) [14,19,20]. However, qPCR overestimates the titer since the DNA copy of the lentiviral RNA genome that is inserted into the host cell genome yields varying expression levels depending on the chromatin region [15]. To overcome this drawback, measurement of the transgene expression at the mRNA level by reverse transcription qPCR (RT-qPCR) is an alternative, but requires a time-consuming RNA extraction step [19]. Another commonly used technique for infectious titer determination is flow cytometry. With this technology the expression of the gene of interest or a reporter gene, such as the green fluorescent protein (GFP) is measured [15]. Using GFP as a reporter gene has the advantage of eliminating staining steps, however, the usage of lentiviral vectors transferring the gene for GFP is limited to *in vitro* and preclinical *in vivo* research. In the case of lentiviral vectors used for therapeutic applications such as CAR-T cell therapy, the expressed CAR construct is detected by a fluorophore-conjugated antibody during flow cytometry analysis or by measuring the mRNA expression of the transgene with RT-qPCR [19,21]. These assay formats are multi-step processes, generally time consuming and results are obtained after four to five days [21,22]. Furthermore, both methods lack the ability for high-throughput sample processing that can be easily implemented at a relatively low cost.

**Fig 1:**
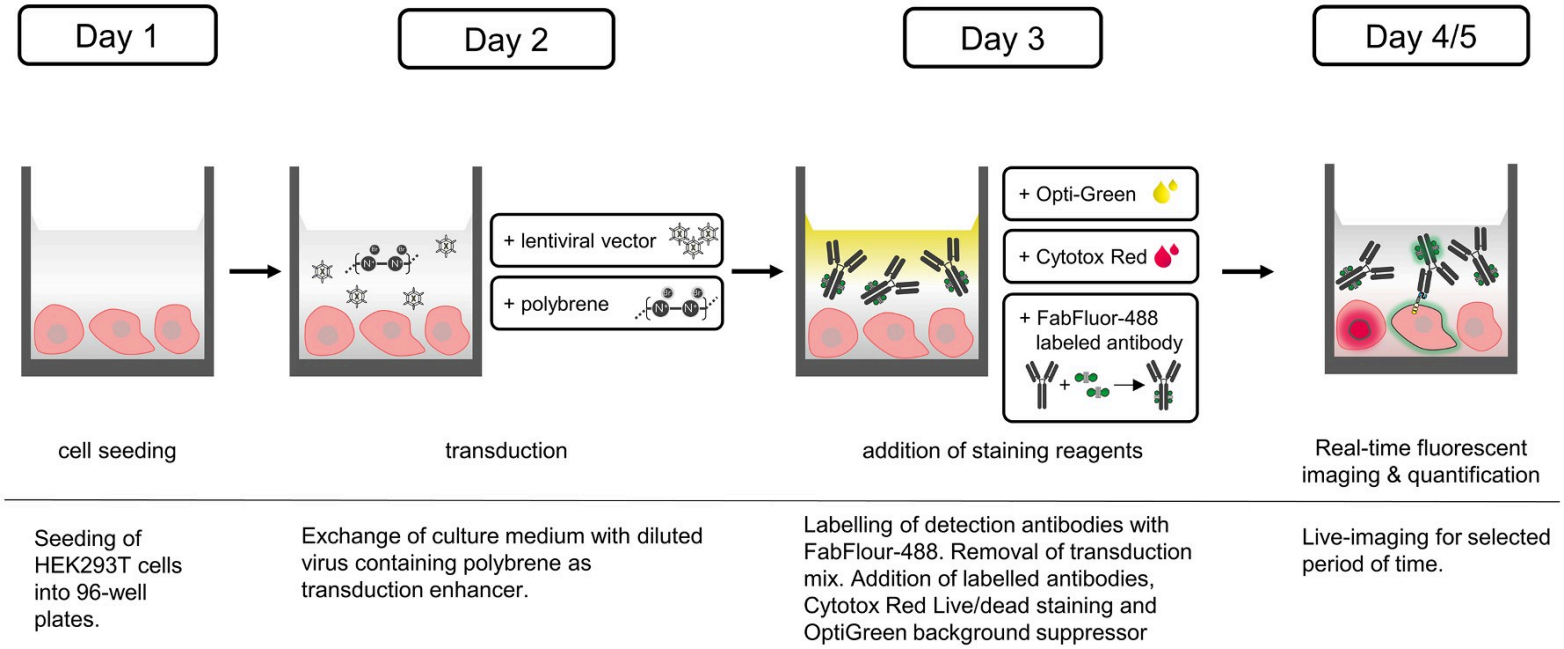
Schematic workflow of the infectious titer assay performed with the Incucyte^®^ S3. HEK293T cells were seeded into a 96-well plate on day 1. The next day, the cells were transduced with diluted lentiviral vector and the transduction enhancer polybrene. 24 h post-infection the transduction mix was removed and replaced by a mixture of staining reagents containing FabFluor-488 labeled anti-FMC63 scFv antibody, Cytotox Red, and Opti-Green background suppressor. Dead cells were stained by Cytotox Red. Infected cells expressing the anti-CD19 CAR were stained by the FabFluor-488 labeled anti-FMC63 scFv antibody. Viable non-infected cells remained unstained. Quantification was performed by real-time fluorescence imaging from days 3-5.

To address the bottleneck of current analytical methods for viral vectors, we developed a new immunological real-time imaging method to quantify the infectious titer of anti-CD19 CAR lentiviral vectors using the Incucyte^®^ S3. The principle of this approach is shown in Fig 1.

**Fig 2:**
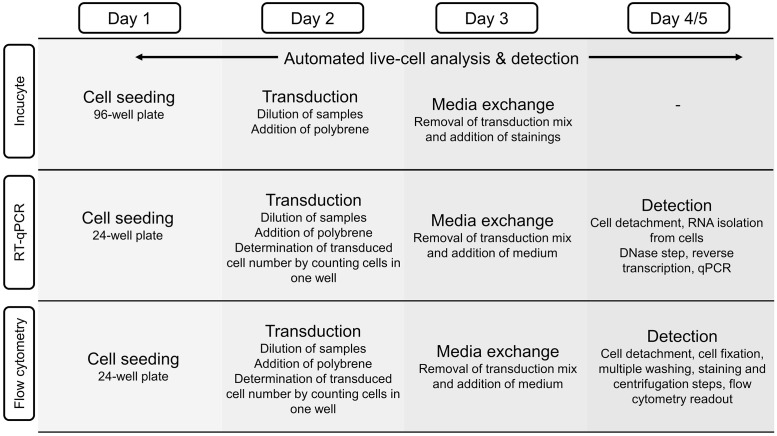
Workflow comparison of lentiviral vector (LV) infectious titer determination methods. One day after cell seeding, cells are transduced with LV and transduction enhancer; measurement of cell number may be required to determine the cell number at time of transduction. On the third day the transduction mix is removed and replaced by growth medium and the staining mixture for the Incucyte^®^ method. While the Incucyte^®^-based assay continually detects transduced cells with no further action required, the flow cytometry and RT-qPCR based assays require intensive handling for the final (endpoint) readout on day 4 or 5.

The introduction of a temporal readout simplified the workflow, increased throughput, and reduced labor. The applicability of the established method for lentiviral vector infectious titer quantification was shown by studying the stability of lentiviral vectors towards external influences. A comparison of the different workflows for infectious LV titer determination, either by flow cytometry, RT-qPCR, or by the newly developed real-time imaging approach is shown in Fig 2.

## Materials and methods

### Lentiviral vector production

Third generation lentiviral vectors were produced by transient transfection of suspension HEK293T/17 SF cells (ACS-4500, ATCC) with four plasmids in an Ambr^®^ 250 Modular bioreactor system (Sartorius). The cultivation parameters were: Temperature of 36.8°C, 30% dissolved oxygen, pH 7.1, and stirring speed 400 rpm. The plasmids and transfection method are described in detail in Labisch *et al* [23].

### Lentiviral vector harvest and clarification

The lentiviral vector was harvested 72 h post-transfection. DENARASE^®^ (c-Lecta) and MgCl_2_ (Carl Roth) were added to the cell culture broth one hour before harvest at a final concentration of 10 U/mL and 2 mM, respectively. After nucleic acid digestion, the lentiviral vector containing cell culture broth was directly clarified with Sartoclear Dynamics^®^ Lab V50 (0.45 μm polyethersulfone membrane version) with 5 g/L diatomaceous earth (Sartorius). The lentiviral vector was aliquoted and stored at -80°C.

### Infectious titer determination using flow cytometry

To quantify the infectious lentiviral vector titer by flow cytometry, adherent HEK293T cells (ACC 635, DSMZ) were infected with serially diluted LV samples and the expression of the EGFRt-transgene-fusion protein was detected. 6·10^4^ cells/well were seeded in 0.5 mL Dulbecco’s modified Eagle medium (DMEM; Thermo Fisher Scientific) + 10% fetal calf serum (FCS; Sigma Aldrich) (v/v) in a tissue culture (TC) treated 24-well plate (Greiner Bio-one). Cells were incubated at 37°C and 5% CO_2_ in a static incubator (Sartorius) for one day. To infect the cells, culture medium was removed, and the cells were transduced by transferring 0.5 mL of diluted virus solution containing 8 μg/mL polybrene (Sigma Aldrich). A negative control of the respective LV batch was analyzed as well. 18 h post infection, the medium was removed and replaced with fresh culture medium. 72 h post infection the expression of the gene-of-interest was analyzed by flow cytometry. First, cells were detached by incubation with 200 μL trypsin-EDTA (Thermo Fisher Scientific) for 5 min at 37°C. The enzymatic reaction was stopped by adding 500 μL culture medium, subsequently the plate was centrifuged at 300 x g for 5 min and the supernatant was removed. Cells were resuspended in 150 μL PBS and transferred to a non-TC 96-well plate with conical bottom (Sartorius). The 96-well plate was centrifuged, and the supernatant was discarded. To discriminate viable and dead cells, 100 μL of a 1:1000 dilution (in PBS) of the fixable fluorescent dye Zombie NIR^™^ (BioLegend) was added to each well and incubated for 10 min in the dark. After another centrifugation step and removal of the supernatant, 100 μL Roti^®^-Histofix 4% (Carl Roth) was added to the cells and incubated for 15 min. The supernatant was removed from the wells after centrifugation and cells were washed with 150 μL PBS. Hereafter, centrifugation of the plate was performed, the supernatant was removed and the cells were incubated for 30 min with an anti-human EGFRt phycoerythrin (PE) conjugated antibody (R&D Systems) at a 1:200 dilution in 40 μL staining buffer (1% Bovine serum albumin (Carl Roth) in PBS). The cells were washed twice with 100 μL staining buffer. After each washing step the plate was centrifuged and the supernatant aspirated. Finally, the cells were resuspended in 40 μL staining buffer and flow cytometry was performed with an iQue ScreenerPlus flow cytometer (Sartorius). The obtained data was analyzed using the integrated ForeCyt 8.0 software. The percentage of EGFRt positive cells of viable single cells was determined. Samples were analyzed in duplicates if not indicated otherwise. The functional lentiviral titer, given in transducing units (TU) per mL, was calculated using the following formula:

$$Infectious\;titer\;=\;\frac{P_{1}\cdot N\cdot D}{V\cdot 100}$$ (1)

Where *P*_*1*_ is the percentage of positive (live) cells, *N* is the number of cells at the time of transduction, *D* is the dilution factor of the LV used for infection, and the transduction volume *V* in mL.

### Infectious titer determination using the Incucyte^®^ S3

For quantification of the infectious virus titer with the Incucyte^®^ S3 (Sartorius), adherent HEK293T cells (ACC 635, DSMZ) were infected with serially diluted LV samples and the expression of the CD19-CAR was measured (Fig 1). 6 x 10^3^ cells were seeded (using Picus^®^ NxT 50–1200 μL, 12-channel, multi-dispense mode, speed: 1, Sartorius) in DMEM + 10% FCS (Sigma Aldrich) in a TC-treated, poly-L-lysine (Sigma Aldrich) coated black 96-well plate with clear bottom (Corning). A positive control for a live/dead cell staining was prepared by adding Triton X-100 (Carl Roth) at a final concentration of 0.005% to the cells. Untreated cells were used as a negative control for the live/dead staining. The cells were incubated at 36.5°C and 5% CO_2_ in the Incucyte^®^ S3 which was located within a static CO_2_ incubator (PHCbi). Each well was analyzed every 2 h, taking four images at a 10x magnification with the phase contrast channel, the red and green fluorescence channel. 24 h after cell seeding the culture medium was removed and replaced by 50 μL diluted LV samples (Picus^®^ NxT 5–120 μL, 8-channel, speed: 1, Sartorius). Polybrene was added at a final concentration of 8 μg/mL to each well. A negative control of the corresponding virus batch was included. Samples were analyzed in triplicates if not indicated otherwise.

To stain the infected cells, the test antibodies were labeled with FabFluor-488 (Sartorius) in a reaction tube before addition to the cells. The anti-FMC63 scFv mouse IgG1 antibody (AcroBiosystems) binding the CD19-CAR construct was used at a final assay dilution of 1:200 and the final concentration of FabFluor-488 was 2.5 μg/mL. A negative isotype control antibody (1 μg/mL) and a positive control anti-transferrin-receptor antibody (3 μg/mL) (R&D Systems), both a mouse IgG1 subtype, were labeled at a 1:3 molar ratio with FabFluor-488. The test antibodies were incubated with Fab-Fluor-488 for 15 min at 37°C in the dark. 24 h post-infection the medium was removed (Picus^®^ NxT 5–120 μL 8-channel, speed: 1, Sartorius) and 150 μL fresh medium with previously prepared staining reagent mixtures, containing FabFluor-488 labeled test antibody, Cytotox Red (Sartorius) and Opti-Green background suppressor (Sartorius) in final concentrations of 250 nM and 0.5 mM, respectively, were added to the cells (Picus^®^ NxT 10–300 μL, 8 channel, speed: 1, Sartorius). Afterwards, the plate was placed into the Incucyte^®^ S3. Imaging was continued as described above for 5 days.

The Incucyte^®^ analysis software was used to determine the phase contrast area as well as the dead cell area, stained by Cytotox Red, and the area of infected cells, indicated by a FabFluor-488 labeling over time. The percentage of positive cells *P*_*2*_ was determined by dividing the FabFluor-488 labeled cell’s area *A*_*FF488*_ by the phase contrast area *A*_*C*_.


$$P_{2}\;=\;\frac{A_{FF488}}{A_{C}}*100$$ (2)

The number of cells at the time of transduction *N* was calculated based on a correlation of the phase contrast area and the number of cells seeded per well determined by an offline cell count analysis. To do so, the samples of different cell counts were analyzed offline with a Cedex HiRes analyzer, followed by seeding of the cells into a 96-well plate and subsequent measurement of the phase contrast area with the Incucyte^®^ S3.


$$N\;=\;\frac{C-b}{m}$$ (3)

*C* is the measured phase contrast area in percent, *b* is the y-intercept, and *m* is the slope of the linear regression of cell count and phase contrast area. With the aid of the determined values from Eqs 2 and 3, the infectious titer was calculated using Eq 1.

The percentage of infected cells can be assessed either accounting for all cells or just for live cells when an additional live/dead cell staining is performed. Cells that are infected but dead, emit a green and red fluorescent signal. These cells are detected by an overlap analysis. The overlap analysis module of the Incucyte^®^ S3 software enables calculation of the infectious titer including positive viable cells. Dead cells emit a red fluorescence signal, due to the Cytotox Red staining and can thereby be excluded from the analysis.

The area of cells that are both infected but dead (*A*_*PD*_) was subtracted from the FabFluor-488 labeled area *A*_*FF488*_ to obtain the area of infected viable cells. This area was divided by the total area of viable cells, which was calculated by subtracting the area of dead cells *A*_*D*_ from the phase contrast area at readout time *A*_*C*_. This gives the percentage of positive viable cells *P*_*3*_.


$$P_{3}\;=\;\frac{A_{FF488}-A_{PD}}{A_{C}-A_{D}}*100$$ (4)

*P*_*3*_ was used to calculate the infectious titer with Eq 1 accounting just for positive viable cells, whereas *P*_*2*_ was used to calculate the titer accounting for all cells.

### Segmentation of phase contrast and fluorescence images

The segmentation adjustment to bias the segmentation between cells and background was set to 1.0. The minimum area was set to 170 μm^2^ which leads to exclusion of smaller objects from the analysis e.g. cell debris. The outlines of the confluence mask were refined by applying a clean-up filter of -2 pixels. HEK293T cells that were infected by the LV and expressed the CAR construct could be detected in the green fluorescence channel after binding of a FabFluor-488 labeled anti-FMC63 scFv antibody. The optimal dilution of the anti-FMC63 scFv antibody was chosen to give maximal intensity without excessive background intensity. The edge split filter was deactivated to enable the determination of the green positive area. The background was subtracted from the images by setting the top-hat filter to the radius of the largest fluorescent object. Objects brighter than the specified threshold were detected in the background-subtracted image. The optimal settings were determined to be a top-hat filter radius of 20 μm, a threshold of 1.3 green calibrated units (GCU), a clean-up filter of -1 pixel, and a minimum area filter of 35 μm^2^. For detection of dead cells, the top-hat filter was set to a radius of 20 μm, and a threshold of 0.3 red calibrated units (RCU) was selected. An area filter of 35–1400 μm^2^ lead to recognition of objects only in the specified size range. The minimum thresholds of the FabFluor-488 mask and the Cytotox Red mask were set according to the positive and negative controls of the FabFluor-488 and Cytotox Red staining, thus false positives and unspecific binding events would not significantly affect the calculated titers. The IgG1 isotype antibody and the matrix control containing no LV gave no FabFluor-488 signal and served as negative controls. The anti-transferrin-receptor IgG1 antibody confirmed successful FabFuor-488 labeling of the antibodies (S1 Fig).

### Lentiviral vector stability study

The lentiviral vector was frozen by placing the samples into a -80°C refrigerator. The samples were thawed rapidly in a water bath at 37°C. The freeze and thaw procedures were repeated for multiple freeze and thaw cycles. To study the infectivity loss upon temperature exposure, the lentiviral vector was incubated at 4°C, 22°C, and 37°C for 24 h, 48 h, and 72 h. An untreated sample served as a negative control. The effect of salt on LV infectivity was analyzed by incubating the LV with 0.2, 0.4, 0.6, 0.8, and 1 M of NaCl in PBS for 1 h at 4°C. Incubation was stopped by diluting to 0.1 M NaCl. A sample without NaCl treatment incubated for 1 h at 4°C served as a negative control. Shear stress caused by pumping was analyzed. A tube (#16, 3.2 mm x 1.6 mm, Watson Marlow) was connected to a peristaltic pump 120U/DV (Watson Marlow) and a reservoir containing 30 mL of the lentiviral vector sample. The sample was pumped at 19.5 mL/min or 78.2 mL/min for 10 min or 30 min in a cycle. A sample incubated for 10 min or 30 min at room temperature served as a negative control. All experiments were performed in triplicates. Each replicate was analyzed in duplicates in the Incucyte^®^ S3.

### Statistical analysis

The statistical significance of between-group differences was evaluated by using unpaired Student’s t-tests (two-tailed) with OriginPro^®^ 2020 (OriginLab). Differences among more than two groups were evaluated by one-way ANOVA with Holm-Sidak post-hoc test for multiple comparisons with SigmaPlot 14.5 (Systat).

## Results

### Segmentation of phase contrast and fluorescence images

**Fig 3:**
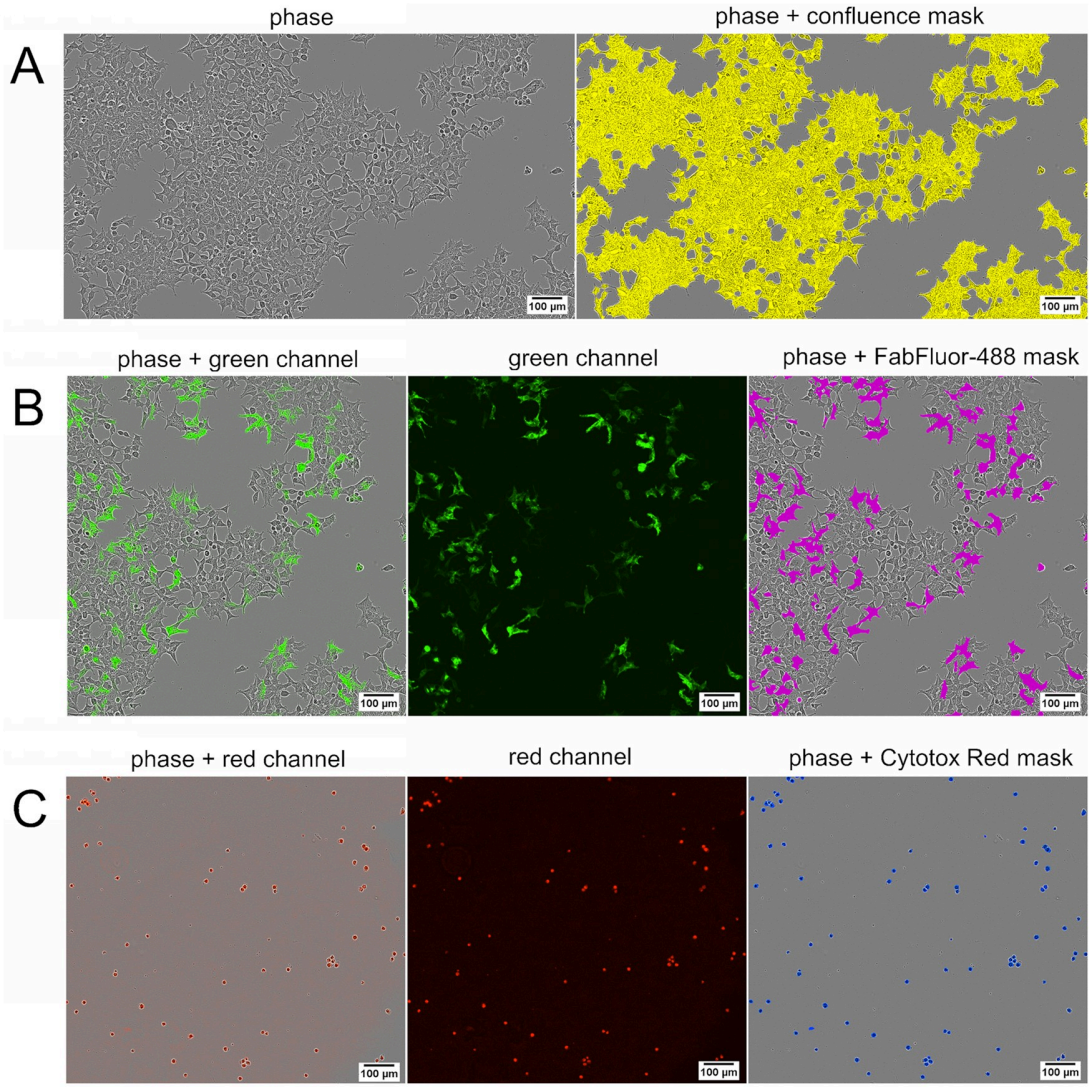
Detection masks for infective titer calculation. (A) Phase contrast image of HEK293T cells (left), merged with phase contrast area analysis mask in yellow (right). (B) HEK293T cells infected with lentiviral vector at a 1:625 dilution. The expressed CAR-construct was stained with an anti-FMC63 scFv antibody labeled with FabFluor-488. Phase contrast image merged with green fluorescence channel (left), background corrected green fluorescence channel (middle), and phase contrast image merged with green fluorescence analysis mask in magenta (right). (C) Live/dead staining positive control cells treated with 0.005% Triton X-100 and stained with Cytotox Red. Phase contrast image merged with red fluorescence channel (left), background corrected red fluorescence channel (middle), and phase contrast image merged with red fluorescence analysis mask in blue (right). All images were taken at 10x magnification.

Part of the development of the infectivity assay for lentiviral vectors with the Incucyte^®^ S3 was defining the analysis parameters for segmentation of phase contrast and fluorescence images. The image analysis software of the Incucyte^®^ S3 allows to process kinetic data from three channels in real-time. High definition phase contrast images were captured and segmented to return a value of total cell area (Fig 3A). The green and red fluorescent images were segmented and yielded area metrics for positively infected cells expressing the CAR construct (Fig 3B) and dead cells respectively (Fig 3C).

### Determination of the linear range and precision of the Incucyte^®^-based infectious titer assay

**Fig 4:**
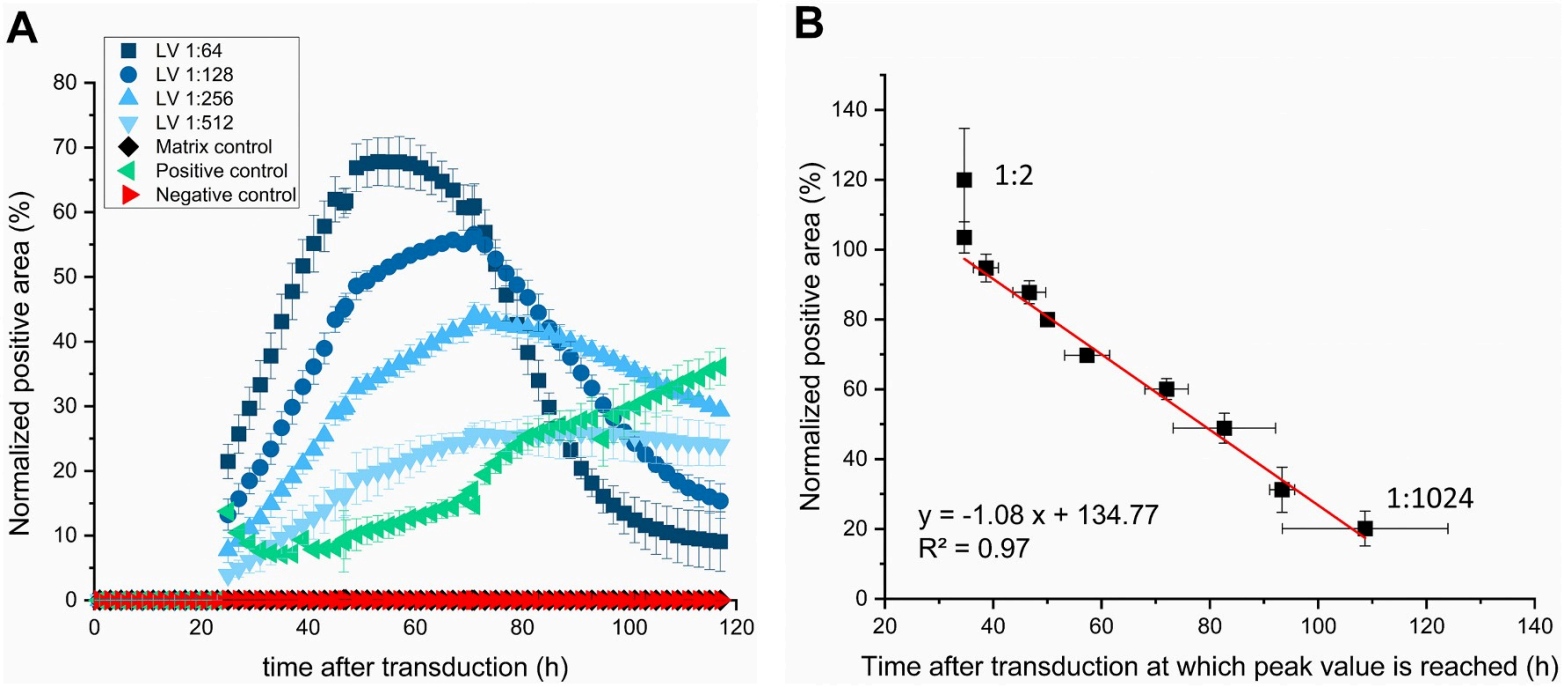
Time course series of normalized positive cell area. (A) The FabFluor-488 positive area was normalized to the phase contrast area over time after transduction. The IgG1 isotype negative control and the lentiviral vector (LV) negative control (matrix) showed no FabFluor488 signal. The anti-transferrin-receptor IgG1 positive control antibody confirmed successful FabFluor488 labeling of the test antibodies. Matrix control overlaps with the negative control. Data represent the mean ± standard deviation of three technical replicates. (B) Normalized FabFluor488 peak value vs. time at which this value is reached. The datapoint in the upper left with the highest normalized positive area represents the 1:2 LV dilution. The other datapoints represent two-fold serially diluted LV up to a dilution of 1:1024 with the lowest normalized positive area and the longest time after which the peak value is reached. Data represent the mean ± standard deviation of three biological replicates.

To identify the linear range of the assay in regard to the normalized positive area of the infected cells, HEK293T cells were infected with serially diluted lentiviral vector. The measurement showed a time-dependent and concentration-dependent effect (Fig 4). For all experiments, a readout time was defined for each curve, which corresponded to the maximum signal over the measured period.

**Fig 5:**
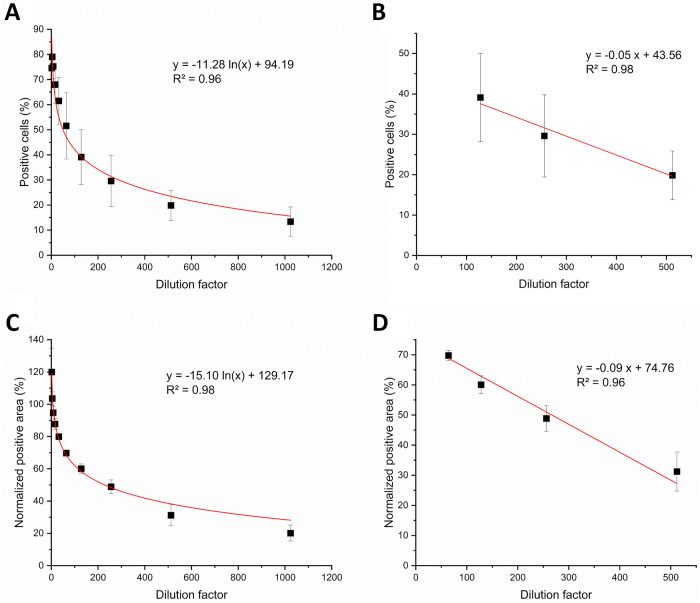
Linear range of the infectious titer assays. The number of positive cells detected via flow cytometry (A & B) and the FabFluor-488 positive area determined with the Incucyte^®^ S3 (C & D) were plotted against dilution factors from a serially diluted lentiviral vector. A logarithmic trend was observed across the whole range of dilutions using the Incucyte^®^ readout with an R^2^ of 0.98 (C) and the flow cytometer readout with an R^2^ of 0.95 (A). A linear dependence was determined for dilutions from 1:64 to 1:512 for the Incucyte^®^ assay with an R^2^ of 0.96 (D) and from 1:128 to 1:512 for the flow cytometer assay with an R^2^ of 0.988 (B). Data represent the mean ± standard deviation of three biological replicates.

To determine the linear range of the flow cytometry and the Incucyte^®^ S3 based methods, cells were transduced with a twofold serial dilution of the LV. With the flow cytometry readout, a logarithmic trend (Fig 5A) across the dilution range was observed and a linear range for dilutions between 1:128 and 1:512 with an R^2^ of 0.98 (Fig 5B). The Incucyte^®^-based method showed a logarithmic trend as well (Fig 5C). A linear range was observed for dilutions between 1:64 and 1:512 with an R^2^ of 0.96 (Fig 5D). According to the linear range of the Incucyte^®^ S3 infectious titer assay, the lower limit of detection (LLOD) and the upper limit of detection correspond to a normalized positive area of 31% and 70%, respectively.

The inter-assay precision between three independently performed assays was analyzed by calculating the standard deviation (SD) and coefficient of variation (CV) (Table 1). The performed assays included the following variations: Day-to-day variation, technician-to-technician variation and batch-to-batch variation of the working cell bank used.

**Table 1:** Inter-assay and intra-assay precision of the Incucyte^®^ infectious titer assay.

	Sample dilution factor	Mean	SD	CV (%)	Z’ factor
**Inter-assay precision** ^a^	2	119.97	14.74	12.29	0.58
	4	103.53	4.46	4.30	0.81
	8	94.75	3.97	4.19	0.81
	16	87.76	3.29	3.75	0.81
	32	79.94	1.77	2.21	0.85
	64	69.76	1.67	2.39	0.84
	128	60.06	3.00	4.99	0.74
	256	48.86	4.28	8.76	0.60
	512	31.23	6.47	20.70	0.15
	1024	20.14	4.98	24.71	-0.12
**Intra-assay precision** ^b^	16	75.45	2.53	3.35	0.89
	128	40.98	3.91	9.53	0.71
	512	20.79	1.85	8.91	0.72

Mean of normalized positive area is given in %, standard deviation (SD), coefficient of variation (CV), and Z’-factor.

^a^ n = 3,

^b^ n = 10.

The Incucyte^®^ S3 infectious titer assay resulted in an inter-assay CV ranging from 2.21% to 12.29% for the LV dilution factors of 2 to 256. The CV should be below 15% for samples above the LLOD. At the LLOD (1:512 dilution) the CV was 20.7%, which is slightly higher than the accepted CV of up to 20% for the LLOD [24]. For LV dilutions below the LLOD, the CV increased. The Z’-factor is a statistical characteristic giving an indication of assay quality in high throughput screening assays and does find applicability in image-based high throughput analysis assays [25]. A Z’-factor between 0 and 0.5 gives a marginal assay and a Z’-factor between 0.5 and 1 gives an excellent assay [26]. The Z’-factor of the inter-assay experiment was ≥ 0.58 for the LV dilution factors between 2 and 256. At the LLOD and below, the Z’-factor was below 0.5. The intra-assay precision was determined for a high, medium and low LV concentration of a distinct LV sample with randomly positioned samples on a 96-well plate with 10 replicates for each LV sample (Table 1). The intra-assay CV was below 9.53% for all sample concentrations. The Z’-factor for the intra-assay experiment was > 0.71 for all samples. The measured values were independent of the position of the samples on the plate.

### Comparison of infective titer calculation approaches and titration methods

**Fig 6:**
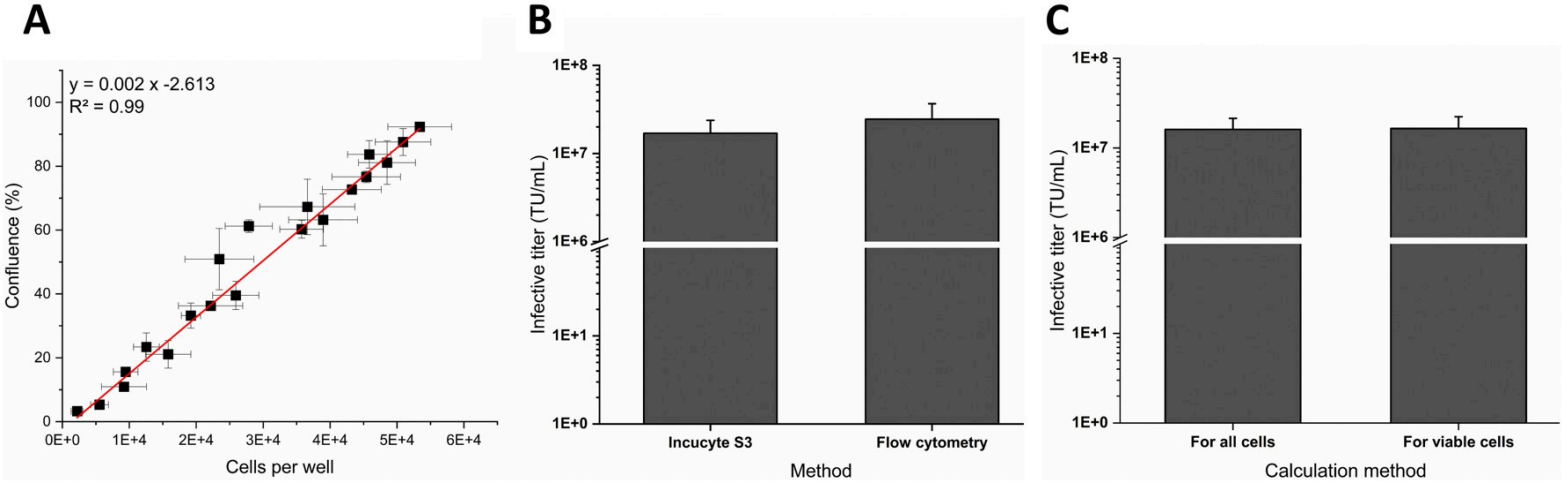
Infectious titer calculation. (A) Correlation of cell count and cell confluence. The cell confluence measured 7 h after cell seeding with the Incucyte^®^ was plotted against the cell count seeded per well as determined by the Cedex HiRes^®^ Analyzer. Mean ± standard deviation, linear regression fit applied (red line) with R^2^ = 0.99. (B) Infective titer for viable cells determined by the Incucyte^®^ S3 and by flow cytometry. (C) Infective titer for three samples of different virus dilutions within the linear range of the Incucyte^®^-based assay calculated either for all cells or for viable cells. Data represent the mean ± standard deviation of three biological replicates.

After characterizing the assay and identifying its linear range, the infectious titer was calculated. To calculate the infectious titer with Eq 1, the number of cells at the time of transduction was determined. Various samples of different cell concentrations were prepared, and the total cell density was measured by the Cedex HiRes^®^ Analyzer (Roche). Cells were seeded in triplicates into a 96-well plate with a density between 2200-53400 cells/well and the phase contrast area was monitored every 30 min over 12 h with the Incucyte^®^ S3. Cells were fully attached after 7 h. The phase contrast area (confluence) 7 h after seeding was plotted against the cell number seeded per well (Fig 6A). The measured confluence showed a linear correlation to the seeded cell number. Linear regression of the measurements resulted in a coefficient of determination (R^2^) of 0.99.

To assess the accuracy of the assay results, the titer of a single lentiviral vector batch was determined by the flow cytometry and by the Incucyte^®^ protocol for three independent biological replicates (Fig 6B). The titers were calculated for dilutions of a lentiviral vector sample that were in the linear range of the respective method. The infective titer obtained by the Incucyte^®^ protocol was 1.70 x 10^7^ ± 0.68 x 10^7^ TU/mL and 2.45 x 10^7^ ± 1.23 x 10^7^ TU/mL for the flow cytometry-based protocol. The functional titers were not significantly different. The infectious titer was calculated either including all cells or viable cells. With the infectious titer assay performed with the Incucyte^®^ a 99.9 ± 0.1% viability of the cells was determined over the whole time course. The infectious LV titers calculated with the two different approaches (Fig 6B) did not differ significantly (unpaired t-test). For this reason, the infective titer was calculated for all cells in further experiments. With the flow cytometry protocol, the cell viability at the time of readout (72 h post-infection) was 88.3 ± 4.4%.

### Application of the newly established infectious titer assay protocol to investigate lentiviral vector stability

**Fig 7:**
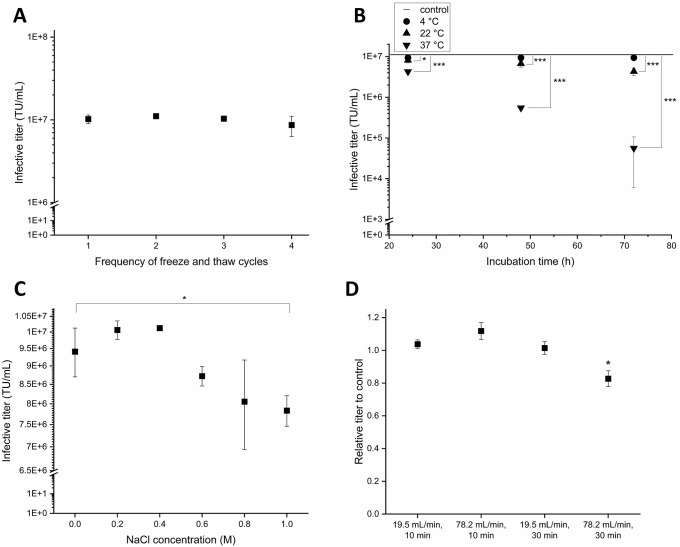
Investigation of external factors on lentiviral vector stability. Assessment of the influence of repeated freeze and thaw cycles (A), storage at different temperatures over time (B), control line represents non-treated LV sample, different sodium chloride (NaCl) concentrations (C) and shear stress induced by a peristaltic pump (D) on lentiviral vector (LV) infectivity. Experiments were performed in biological triplicates. Data represent the mean ± standard deviation. P-values are indicated as follows: * p ≤ 0.05; ** p ≤ 0.01; *** p ≤ 0.001.

During downstream processing (DSP) the LV can be exposed to different physical conditions. It is, therefore, necessary to investigate which process parameters affect the infectious titer. According to current literature it is known that lentiviral vector is prone to damage and loss of infectious titer by several external factors, including temperature, pH, conductivity and shear stress [27]. In this study we analyzed the impact of several physical conditions on the infectivity of a vesicular stomatitis virus glycoprotein (VSV-G) pseudotyped HIV-1 derived LV (Fig 7). Several freeze and thaw cycles were performed, and the LV was exposed to different temperatures for different incubation times. Moreover, the impact of exposure to salt and shear stress by a peristaltic pump was analyzed.

The LV samples were thawed rapidly in a water bath at 37°C and then stored for 1 day at -80°C between each freeze and thaw cycle. The LV maintained its activity over four freeze and thaw cycles (Fig 7A). Thermostability is an important aspect to consider when manufacturing the LV. The purification process is mostly performed at room temperature because it is more economical. Process operations at lower temperatures would require cooling of the instrument and the LV feed or working in a cold room. Stability of LV at a temperature of 37°C are mainly of interest during its upstream processing or during the workflow of an infectious titer assay, where cells are incubated with the virus at 37°C for about 24 h. In our study we identified that the LV infectious titer was not significantly affected by storage at 4°C over an incubation period of 72 h (Fig 7B). At 22°C and 37°C the LV infectivity decreased significantly for all investigated incubation times. LV half-lives were calculated from a 4-point kinetic experiment. At room temperature, a decay of infectious titer over time was observed with a half-life of 75.3 ± 12.1 h. The LV half-life at 37°C was 37.0 ± 11.8 h.

Sodium chloride is often used for elution of the virus during its purification by ion exchange chromatography, with typical concentrations in a range between 1 M and 1.5 M [28,29]. In our study, the infectious titer decreased between 0.6 and 1 M NaCl (Fig 7C) after incubation for 1 h at 4°C. When compared with the no NaCl control sample, the samples exposed to 1 M showed a significant decrease of LV infectivity by 17%.

Shear stress induced by continuously pumping the virus through a tube with a peristaltic pump reduced LV infectious titer significantly by 17% when applying a flow rate of 78.2 mL/min for 30 min (Fig 7D). A shorter time period or lower flow rate did not significantly reduce LV infectivity. A flow rate of 78.2 mL/min applied for the duration of 10 min corresponds to a 78-fold transport of one milliliter through the pump. Within a duration of 30 min, 1 mL is transported 20 times (at flow rate of 19.5 mL/min) or 26 times (at a flow rate of 78.2 mL/min) through the pump, within a duration of 10 min 1 mL of virus is transported 7 times (at 19.5 mL/min) through the pump.

## Discussion

### Immunological real-time imaging approach for infectious titer determination

We developed an immunological real-time imaging protocol for CAR-based lentiviral vector infectious titer determination. The extracellularly expressed protein is bound and stained via labeled antibodies enabling the quantification of lentiviral vectors used for CAR-T cell therapies. The presented protocol is not limited to lentiviral vectors and could be performed with other viral vectors that transfer a gene of interest coding for an extracellular protein or a cell surface receptor, but clearly finds its limitation for genes of interest, that do not code for a receptor or protein located on the cell surface. Moreover, the FabFluor label system is currently applicable for IgG1, IgG2a and IgG2b with a mouse Fc part. For research and development applications, viral vectors transferring the gene for GFP are frequently used [15]. The infectious titer determination for GFP-based viral vectors can be performed with the presented protocol by replacing the staining step by a simple media exchange. Real-time imaging techniques have previously been used to assess the transduction efficiency based on the expression of GFP or other fluorescent reporter proteins by determining the percentage of positive cells or the green object count [30–36]. In contrast to these methods, the workflow presented in this study allows for the determination of the infectious viral titer which is an important value for quality control in upstream and downstream processes of viral vectors. Stewart *et al*. used the Incucyte^®^ for infectious titer determination of hepatitis C virus by an endpoint measurement after fixation and staining [37]. The evolution of the Incucyte^®^ platform and associated software and reagents has enabled a kinetic approach for titer determination described in our article. To our knowledge, detection of transduced cells expressing a cell surface receptor with a real-time live-cell analysis system to determine infectious viral titers has not been published yet. For accurate titer calculation, the number of cells at the time of transduction must be precisely determined. For flow cytometry protocols the cell number is often determined by detaching the cells of one well before transduction. The measured cell count is then assigned to all wells. A small variance of the cell count between different wells is likely to occur. It is more exact to measure the cell count for each well, which is not possible with flow cytometry or a PCR-based method. For our protocol it was not possible to use the Cell-by-Cell analysis software module of the Incucyte^®^, due to the low cell boundary contrast of HEK cells. The use of this analysis module is possible for other cell types with a higher cell boundary contrast. This would allow to determine the number of cells at time of transduction and the number of positive cells. We addressed this problem and correlated an off-line measured cell count and the phase contrast area detected by the Incucyte^®^ for a precise cell count determination for individual wells. The correlation of phase contrast area and cell count can be quickly performed for any cell line used for the infectious titer assay. Besides, we expect limitations when working with suspension cells. Suspension cells could be used but require an optimization of plate coating and pipetting steps to avoid loss of cells.

It is good scientific practice to determine the infectious lentiviral titer for viable cells only. In our study using HEK293T as target cells, the difference between the infectious titer determined from only viable cells versus all cells was not significant. This can be explained by the observed high cell viability. The high viability during the Incucyte^®^ based assay is due to the gentle protocol, which does not require any centrifugation, washing, or fixation steps that are usually performed during the flow cytometry protocol. With flow cytometry, generally a lower cell viability during the readout was detected. We assume this is due to the handling of the cells according to the protocol.

For accurate infectious titer calculation, the LV sample needs to be diluted such that the percentage of positive cells is within the linear range of the respective titration method. The infective titer protocol based on the Incucyte^®^ had a broader linear range compared to the flow cytometry protocol. The Incucyte^®^-based protocol showed good inter-assay and intra-assay precision and an excellent Z’-factor for the linear range. The calculated infective titers determined by the Incucyte^®^ had a smaller standard deviation compared with the flow cytometry method, meaning that a greater variation between replicates was observed for the flow cytometry protocol. Although statistical analysis indicated that the calculated titers do not significantly differ between the two methods, we consider the determined titer with the Incucyte^®^ to be more accurate for the reasons outlined above.

The major advantages of the Incucyte^®^ approach are the simple protocol and the temporal readout. The operator can track the percentage of transduced cells whilst the experiment is running and decide for the optimal readout time. Flow cytometry and qPCR rely on an endpoint measurement where the optimal readout timepoint can be easily missed. We have observed that after reaching a maximum, the normalized FabFluor-488 area decreases. Cell surface receptor-mediated antibody internalization could be a reason for a decreasing normalized FabFluor-488 area [38]. The integrated FabFluor-488 intensity over time showed an equivalent time course, indicating that the signal decreases after reaching a maximum (S2 Fig). Upon internalization of the antibody, the FabFluor-antibody complex emits little or no fluorescence due to the lower intracellular pH. Microscopic analysis showed that cell clusters, that were fully detected by the FabFluor analysis mask at the time of the peak intensity, were not fully detected by the mask at later time points due to lower fluorescence intensity in some areas (S3 Fig). This also supports the hypothesis of antibody internalization.

For the operator, the real-time imaging approach means a significant reduction in workload, as fixation, multiple washing, staining, and centrifugation steps, that are typically performed for a flow cytometry based readout [23], are not required. These steps are the rate-limiting steps of the flow cytometry protocol and severely constrain the number of samples that can be analyzed within one week. Automation of the protocol by using a pipetting robot is hardly possible due to the need for multiple centrifugation steps and the risk of aspirating the cell pellet. Furthermore, the investment and footprint of a pipetting robot is high. The handling time was significantly reduced with the Incucyte^®^ real-time imaging approach, eliminating the need for fixation, washing and centrifugation steps. Thereby, a higher throughput was enabled with the possibility to measure up to six 96-well plates simultaneously within one week.

### Virus stability study

We investigated factors affecting lentiviral vector stability. In an experiment to evaluate the effect of repeated freezing and thawing of lentiviral vectors, no loss of infectious titer was observed over four cycles. This allows for LV being stored between DSP steps at -80°C for later analytical testing. In contrast in a comparable study using VSV-G pseudotyped LV produced by TE671 cells an infectivity reduction by freeze and thaw cycles with a half-life of 3.8 cycles was observed. The same study reported half-lives of 10.4 h at 37°C, approx. 50 h at 20°C and 200 h at 4°C [39]. The half-lives at 37°C and 20°C are shorter than the ones determined in our study with 37 h at 37°C and 75 h at 22°C. Dautzenberg *et al*. reported a half-life of 35 h at 37°C for a VSV-G pseudotyped LV produced in adherent 293T cells [40], which is similar to the half-life determined in our study. An aspect to consider is the buffer in which the LV is formulated. The half-lives determined by Dautzenberg *et al*. are greater than those reported by Higashikawa and Chang. This may be due to the different buffer conditions used, as this is consistent with the reported increase in thermostability of lentiviral vectors in the presence of FCS [41]. The LVs used in our study and by Dautzenberg *et al*. were derived from similar cell lines, whereas Higashikawa and Chang did not use a HEK293-based cell line. LV particles produced with different cell lines have a distinct viral envelope constituents profile depending on their producer cell line. The viral envelope composition was reported to affect LV infectivity [42,43]. Besides the formulation aspect, this may be an explanation for greater susceptibility of lentiviral vectors to thermal exposure and freeze and thaw cycles.

We observed an infectivity loss of 17% after incubation with 1 M NaCl for 1 h, which is consistent with the data of Zimmermann *et al*. who determined an infectivity loss of 16% of VSV-G pseudotyped LV after incubation with 1 M NaCl for 1 h [44]. The effect of salt on another retrovirus was analyzed by Segura *et al*. A VSV-G pseudotyped moloney murine leukemia retrovirus (MLV) lost 50% infectivity after 1 h incubation with 1 M NaCl [45]. This highlights limited comparability of different retroviral vectors, even though they have the same envelope protein. The observed loss of infectious lentiviral titer due to exposure to high salt concentrations corresponds to the low recoveries reported for anion exchange chromatography during DSP [27].

Peristaltic pumps are frequently used during DSP, e.g. during clarification when the virus suspension is pumped through a filter. The number of passages of the virus suspension through the pump was observed to be the critical factor for infectivity reduction. When 1 mL was transported either 7 or 26 times through the pump, the infectivity was not affected independent of the applied flow rate. However, after 78 circulation cycles the infectivity was reduced significantly by 17%. A limitation of the circulation cycles of the viral vector through the system should therefore be considered. The negative impact of shear forces on LV infectivity is often mentioned as an important factor [46]. The stability of VSV-G pseudotyped LV towards shear forces has been previously analyzed during ultracentrifugation. About 54% of the infectious titer was recovered after ultracentrifugation demonstrating the effect of shear forces [47]. Ruscic *et al*. [18] investigated potential losses of RDpro pseudotyped LV infectivity through shear stress applied by the chromatography device by letting the LV pass through the system without capture. They have reported no significant loss of infectious LV in the chromatography system, consequently, no impact of shear forces generated in the system was observed. Control experiments are indispensable to estimate the LV loss caused by the system itself.

The LV stability study, which resulted in a total of 78 samples emphasizes the major advantage of the newly established Incucyte^®^-based infectious titer assay in terms of sample throughput. All samples were analyzed within one week in technical duplicates. The flow cytometry protocol was not performed because the analysis of the samples would have taken 4-7 weeks. Due to the high amount of labor needed to perform infectious titer assays with methods established so far, stability studies were rarely performed and published in the literature. The Incucyte^®^ protocol enables the operator to quickly perform infectious titer assays, resulting in the ability to test even more conditions during process development or to assess the most optimal formulation of the final virus product in reduced time.

In conclusion, we developed an immunological real-time imaging method to quantify the infectious titer of anti-CD19 CAR lentiviral vectors using the Incucyte^®^ S3. The newly established method decreased labor profoundly and thus increased throughput. The real-time imaging and analysis capabilities of the Incucyte^®^ enable an accurate and precise measurement of the infectious LV titers. The broad linear detection range of this method is advantageous and is well suited for analyzing a variety of samples, such as for virus stability studies.

## Supporting information

S1 Fig Positive and negative controls of the staining protocol. Top row: Phase contrast image of HEK293T cells merged with green channel. Bottom row: Phase contrast image merged with FabFluor-488 mask in magenta. Left column: Anti-transferrin-receptor IgG1 antibody labeled with FabFuor-488 as a positive control. Middle column: IgG1 isotype antibody labeled with FabFuor-488 as a negative control. Right column: Matrix control containing no lentiviral vector with anti-FMC63 scFv antibody labeled with FabFluor-488. All images were taken at 10x magnification.
(TIF)

S2 Fig Total FabFluor-488 integrated intensity of HEK293T cells infected by several lentiviral vector dilutions over time. FabFluor-488 fluorescence intensity was analyzed on three independently infected plates (A-C). Lentiviral vector (LV) dilutions were between 1:2 and 1:1024. Low virus dilutions showed a higher integrated intensity, its peak is reached earlier, and the signal decreases earlier compared to high virus dilutions. The negative controls (matrix and medium control) gave no signal.
(TIF)

S3 Fig FabFluor-488 mask over time. HEK293T cells after infection with a 1:2 diluted lentiviral vector and staining 24 h post-infection. Phase contrast image were merged with FabFluor-488 mask, shown in magenta. Yellow arrow indicates a fully detected cell cluster 66 h post-infection that is not fully detected by the FabFluor-488 mask at later time points (90 h and 98 h post-infection). Normalized positive areas were 90.5% (A), 68.8% (B) and 63.3% (C) for the representative images.
(TIF)

S1 Table All data used for figures, tables, and statistical analyses. (XLSX)
