# Supplementary figures and images for: Infectious titer determination of lentiviral vectors using a temporal immunological real-time imaging approach
Source: PLoS One. 2021 Jul 15;16(7):e0254739. doi: 10.1371/journal.pone.0254739 (PMC8281989; doi:10.1371/journal.pone.0254739)

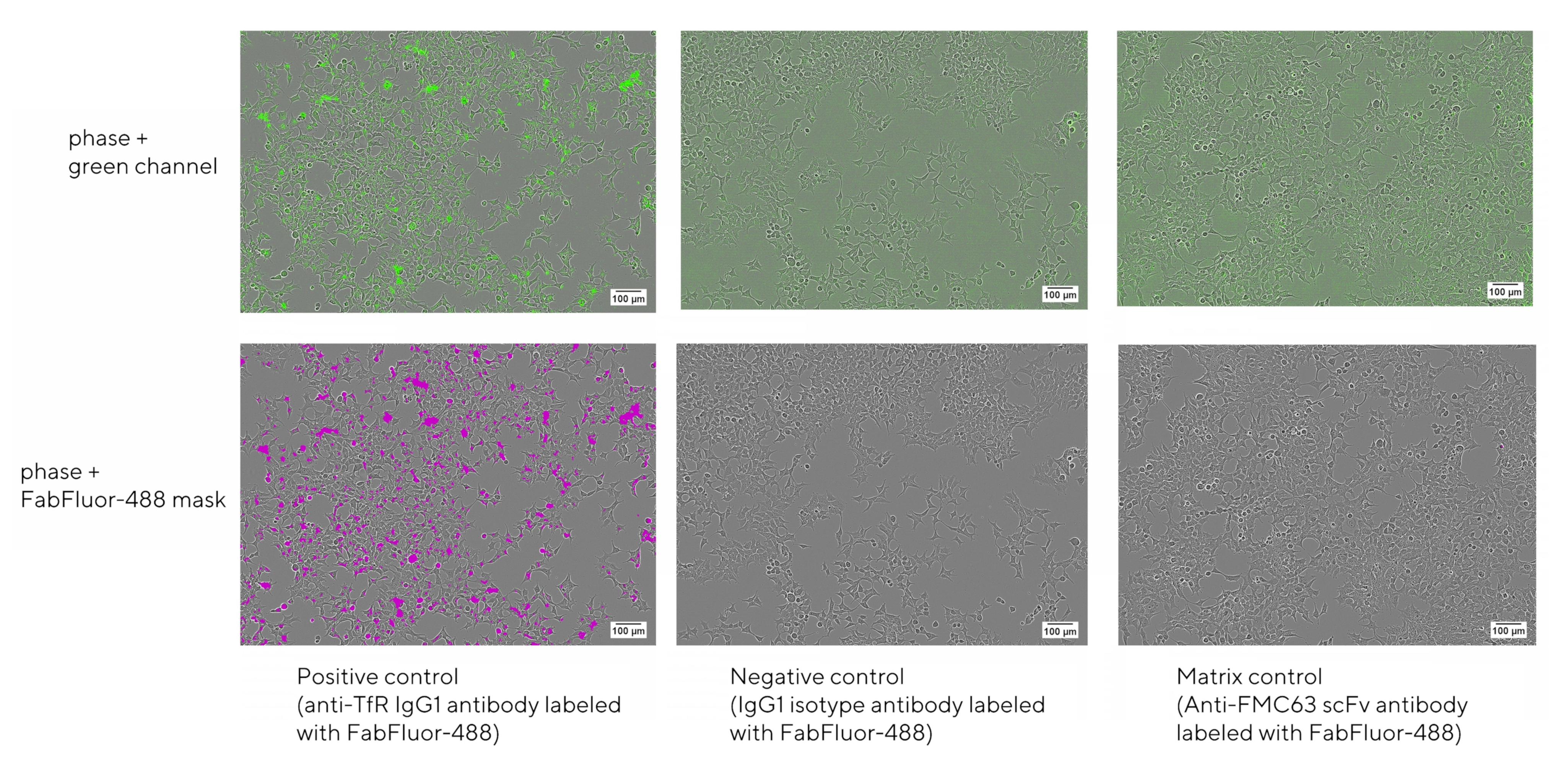

Supplement: S1 Fig — Top row: Phase contrast image of HEK293T cells merged with green channel. Bottom row: Phase contrast image merged with FabFluor-488 mask in magenta. Left column: Anti-transferrin-receptor IgG1 antibody labeled with FabFuor-488 as a positive control. Middle column: IgG1 isotype antibody labeled with FabFuor-488 as a negative control. Right column: Matrix control containing no lentiviral vector with anti-FMC63 scFv antibody labeled with FabFluor-488. All images were taken at 10x magnification. (TIF) [file pone.0254739.s001.tif]

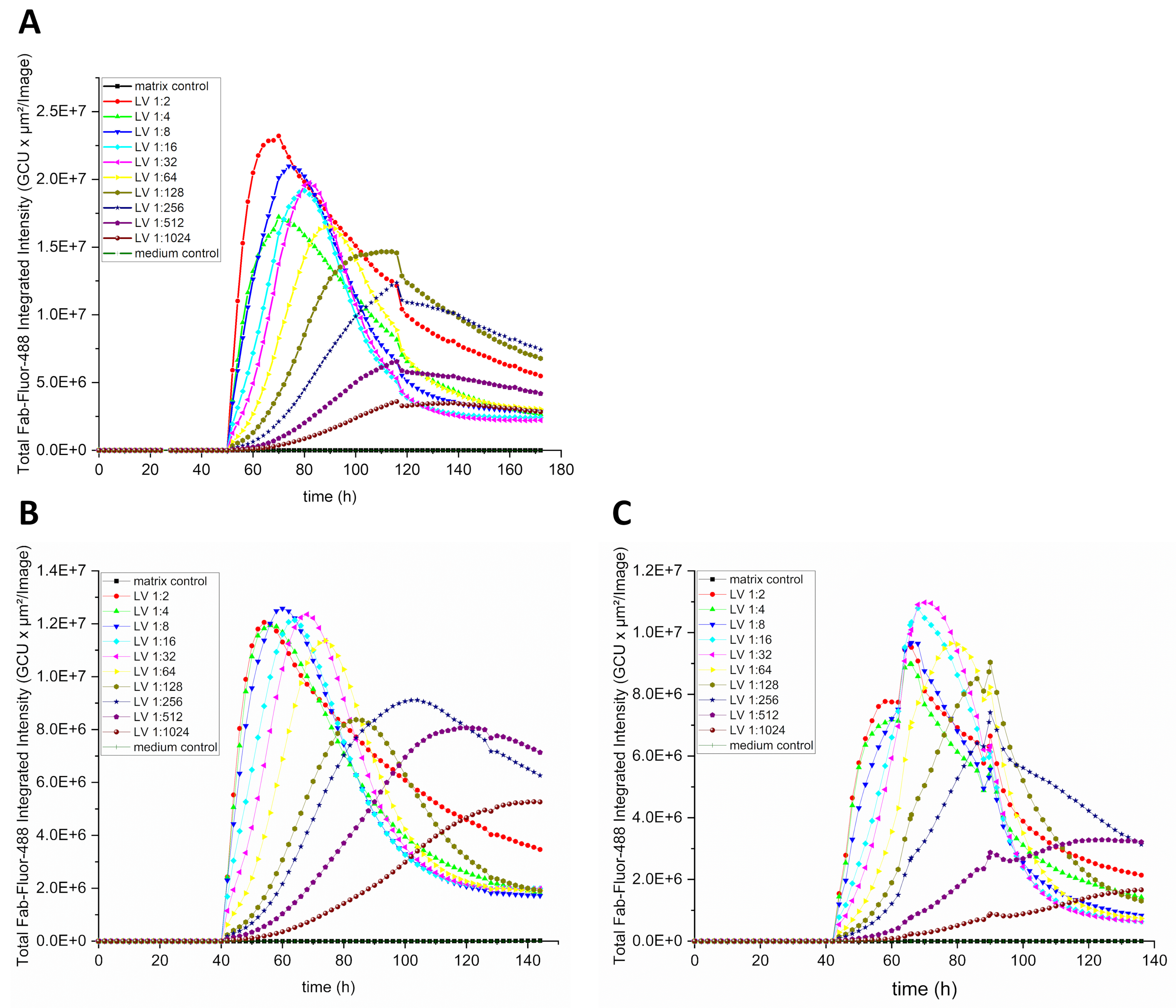

Supplement: S2 Fig — FabFluor-488 fluorescence intensity was analyzed on three independently infected plates (A-C). Lentiviral vector (LV) dilutions were between 1:2 and 1:1024. Low virus dilutions showed a higher integrated intensity, its peak is reached earlier, and the signal decreases earlier compared to high virus dilutions. The negative controls (matrix and medium control) gave no signal. (TIF) [file pone.0254739.s002.tif]

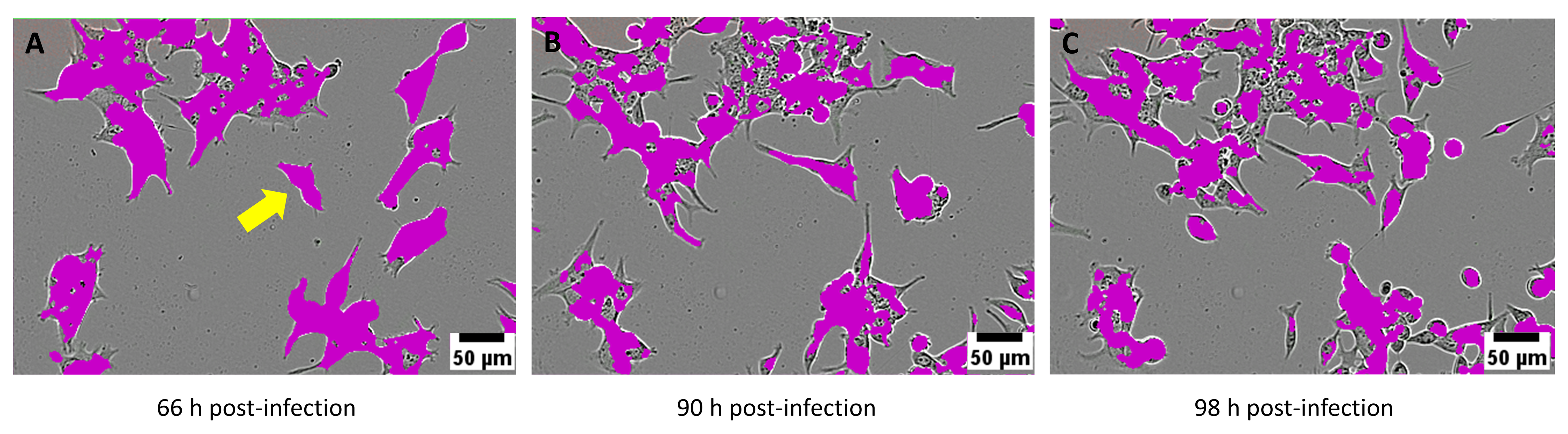

Supplement: S3 Fig — HEK293T cells after infection with a 1:2 diluted lentiviral vector and staining 24 h post-infection. Phase contrast image were merged with FabFluor-488 mask, shown in magenta. Yellow arrow indicates a fully detected cell cluster 66 h post-infection that is not fully detected by the FabFluor-488 mask at later time points (90 h and 98 h post-infection). Normalized positive areas were 90.5% (A), 68.8% (B) and 63.3% (C) for the representative images. (TIF) [file pone.0254739.s003.tif]
